# A Specific *IL6* Polymorphic Genotype Modulates the Risk of *Trypanosoma cruzi* Parasitemia While *IL18*, *IL17A*, and *IL1B* Variant Profiles and HIV Infection Protect Against Cardiomyopathy in Chagas Disease

**DOI:** 10.3389/fimmu.2020.521409

**Published:** 2020-10-22

**Authors:** Alexandra Gomes dos Santos, Elieser Hitoshi Watanabe, Daiane Tomomi Ferreira, Jamille Oliveira, Érika Shimoda Nakanishi, Claudia Silva Oliveira, Edimar Bocchi, Cristina Terra Gallafrio Novaes, Fatima Cruz, Noemia Barbosa Carvalho, Paula Keiko Sato, Edite Hatsumi Yamashiro-Kanashiro, Alessandra Pontillo, Vera Lucia Teixeira de Freitas, Luiz Fernando Onuchic, Maria Aparecida Shikanai-Yasuda

**Affiliations:** ^1^ Department of Infectious and Parasitic Diseases, Faculdade de Medicina, University of São Paulo, São Paulo, Brazil; ^2^ Department of Medicine, Divisions of Molecular Medicine and Nephrology, Faculdade de Medicina, University of São Paulo, São Paulo, Brazil; ^3^ Laboratory of Immunology (LIM 48), Hospital das Clínicas, Faculdade de Medicina, University of São Paulo, São Paulo, Brazil; ^4^ Heart Institute, Hospital das Clínicas, Faculdade de Medicina, University of São Paulo, São Paulo, Brazil; ^5^ Division of Infectious Diseases, Hospital das Clinicas, Faculdade de Medicina, University of São Paulo, São Paulo, Brazil; ^6^ Instituto de Medicina Tropical, University of São Paulo, São Paulo, Brazil; ^7^ Departament of Immunology, Instituto de Ciências Biomédicas (ICB), University of São Paulo, São Paulo, Brazil

**Keywords:** *T. cruzi* parasitemia, cardiomyopathy, Chagas disease, HIV, *IL1 B*, *IL6*, *IL17 A* and *IL18* polymorphisms

## Abstract

**Background:**

Chagas disease caused by *Trypanosoma cruzi* (*T. cruzi*) affects approximately six million individuals worldwide. Clinical manifestations are expected to occur due to the parasite persistence and host immune response. Herein we investigated potential associations between *IL1B*, *IL6*, *IL17A*, or *IL18* polymorphism profiles and cardiomyopathy or *T. cruzi* parasitemia, as well as the impact of HIV infection on cardiopathy.

**Methods:**

Two hundred twenty-six patients and 90 control individuals were analyzed. *IL1B* rs1143627 T>C, *IL6* rs1800795 C>G, *IL17A* rs2275913 G>A, *IL18* rs187238 C>G, and *IL18* rs1946518 C>A SNVs were analyzed by real-time PCR and *T. cruzi* parasitemia by PCR.

**Results:**

Our data revealed association between a cytokine gene polymorphism and parasitemia never previously reported. The *IL6* rs1800795 CG genotype lowered the risk of positive parasitemia (OR = 0.45, 95% CI 0.24–0.86, P = 0.015). Original findings included associations between *IL17A* rs2275913 AA and *IL18* s1946518 AA genotypes with decreased risk of developing cardiomyopathy (OR = 0.27, 95% CI 0.07–0.97, P = 0.044; and OR = 0.35, 95% CI 0.14–0.87, P = 0.023, respectively). *IL18* rs1946518 AA and *IL1B* rs1143627 TC were associated with reduced risk for cardiomyopathy severity, including NYHA (New York Heart Association) class ≥ 2 (OR = 0.21, 95% CI 0.06–0.68, P = 0.009; and OR = 0.48, 95% CI 0.24–0.95, P = 0.036, respectively) and LVEF (left ventricular ejection fraction) <45% for *IL18* rs1946518 AA (OR = 0.22, 95% CI 0.05–0.89, P = 0.034). A novel, unexpected protective effect of HIV infection against development/progression of cardiomyopathy was identified, based on a lower risk of developing cardiopathy (OR = 0.48, 95% CI 0.23–0.96, P = 0.039), NYHA class ≥ 2 (OR = 0.15, 95% CI 0.06–0.39, P < 0.001), and LVEF < 45% (OR = 0.03, 95% CI 0.00–0.25, P = 0.001). Digestive involvement was negatively associated with NYHA ≥ 2 and LVEF < 45% (OR = 0.20, 95% CI 0.09–0.47, P < 0.001; and OR = 0.24, 95% CI 0.09–0.62, P = 0.004, respectively).

**Conclusions:**

Our data support a protective role of *IL17A* AA, *IL18* AA, and *IL1B* TC genotypes against development/progression of cardiomyopathy and a modulatory effect of the *IL6* CG genotype on the risk of parasitemia in Chagas disease. Notably, HIV infection was shown to protect against development/progression of cardiopathy, potentially associated with a synergistic effect of HIV and highly active antiretroviral therapy (HAART), attenuating a Th1-mediated response in the myocardium. This proposed hypothesis requires confirmation, however, in larger and more comprehensive future studies.

## Introduction

Chagas disease, a potentially life-threatening illness caused by the protozoan *Trypanosoma cruzi* (*T. cruzi*) and endemic in Latin America, affects approximately 6 million individuals worldwide. The natural course of the infection comprises acute and chronic phases. A clinically relevant acute form is observed in only 1/30 infected people, including fever, adenopathy, palpebral or legs edema and/or hepatosplenomegaly and, less often, myocarditis and meningoencephalitis. In the chronic phase, on the other hand, ~30% of the patients develop the cardiac form, expressed as arrhythmia, heart failure, and/or thromboembolic events. The chronic phase also includes a digestive form, which comprises dysphagia and/or constipation in 10–15% of the cases and, in ~5% of them, a cardio-digestive form. The indeterminate form, in turn, represented by asymptomatic patients with normal electrocardiogram and thorax and digestive tract X-rays, constitutes the most frequent chronic form, affecting 60–70% of the referred group (1, 2).

Chagas cardiopathy is a major cause of cardiovascular-related mortality within the 30–50-year age range in Latin American endemic areas (1, 3). Previous studies have recognized the fundamental role of host features in Chagas disease pathophysiology, revealing different immunological profiles in patients affected and not affected by cardiopathy (4). While the development of cardiopathy has been linked to a Th1 response in heart tissue associated with mononuclear cell infiltrate, cardiomyocyte damage, and fibrosis, a Th2 response predominates in the indeterminate form (5, 6). Reports of higher levels/expression of IL-6, IL1β, TNFα, and IFNγ in the cardiac form (7, 8) and IL6 and/IL1β or C-reactive protein in the cardiac and/or cardio-digestive forms (9, 10) support a potential role for such cytokines in the adaptative immune response at heart tissue damaging process. IL6 and IL1β could be considered, therefore, a marker of cardiac lesion. In contrast, the finding of higher plasma levels of IL17A directly associated with better systolic function in the indeterminate form suggests a protective role for this cytokine against the development of clinically relevant chronic Chagas cardiopathy, despite inverse association with diastolic performance indices (11, 12).

Previous studies in patients with indeterminate and cardiac forms suggested a role for T reg cells in the modulation of inflammatory immune responses associated with the cardiac form (6, 13). Increased number of Foxp3+, CD25+, CD4+ T cells, T reg cells that stimulate secretion of IL-17 A, IL-10, and granzyme B, was observed in the indeterminate form, in association with less inflammation and better myocardium function. In contrast, more pronounced immune responses to the parasite, associated with higher levels of inflammatory cytokines and myocardial lesion, correlated with higher densities of IL6+, IFNγ+, TNFα+, CTLA4+ T reg cells. Such findings suggest a role for T reg cells in controlling the exacerbated immune response and morbidity as well as mortality in *T. cruzi* infection, likely by stimulating Th17 cells and modulating killing effector cells (14).

An important role in the control of cardiac inflammatory lesions and remodeling has been proposed for IL18 (15, 16). This cytokine stimulates IFNγ expression independently of IL12, allowing an IL18-IFNγ cross-talk at the myocardium level during *T. cruzi* infection (17). IL18 upregulation and correlation between IL18 messenger RNA (mRNA) and IFNγ expression have in fact been reported in human Chagas heart tissue (6, 18).

*T. cruzi* parasitemia is another major factor implicated in the progression of chronic Chagas cardiopathy, even if intermittent and low at this disease stage (19–22). Available reports, however, are controversial on this point (23–25). Cytokines have also been shown to control *T. cruzi* parasitic burden and myocardial inflammatory lesions in both chronic and acute phases of murine infection. In chronic infection induced with the Colombian strain in *IL18*-KO mice, a significant reduction in the myocardium parasitic load was observed (26). Acute infections of *IL17A*-KO mice, on the other hand, were associated with lack of activation of immune-related cells that are critical for the killing of *T. cruzi* (27) and with increased mortality and higher-degree myocarditis. Variable parasitic loads were detected, however, depending on the mouse line and the parasite strain (27, 28). This finding unraveled a protective effect of IL17 A, evinced by the association between better cardiac function and higher plasma levels of IL17A in patients without cardiomyopathy (11, 12). In addition, in acute murine infection IL6 was shown to drive the survival of *T. cruzi* infected cardiomyocytes. IL6-KO mice lack the innate immunity crucial for host survival and rapidly die with high levels of IL1 β and inflammatory products, a process reverted by adding recombinant IL6 (29). This finding suggests a critical role for IL6 in modulating inflammatory lesions. Due to the modulating effect of IL6 on Chagas disease cardiomyopathy, the data on human disease likely represent a less severe degree of this inflammation (7, 10).

In immunosupressed patients with HIV infection, elevated IL18 serum concentrations have been reported to influence both Aids and the course of associated infections (30). Interestingly, whereas Chagas disease reactivation does not occur in immunocompetent subjects, this illness natural history is changed in HIV infected patients, being associated with increased morbidity and mortality (31).

As part of the genetic assessment of cytokine roles in Chagas disease, single nucleotide variants (SNVs) associated with various cytokine genes have been previously analyzed in *T. cruzi* infection and cardiopathy progression. No significant association involving the *IL6* SNV rs1800795 −174 C>G was identified in Colombia (32), in a context where the G allele has been associated with higher IL6 levels (33, 34) and patients with heart Chagas disease have been found to present higher IL6 levels (7, 10). Positive findings, in turn, included association between increased risk of cardiomyopathy and the CT haplotype for the *IL1B −*31 T>C and +3954 C>T (rs1143634, synonymous coding sequence variant) polymorphisms in Colombia (35). The possible proinflammatory nature of this haplotype (35) is unclear, since the *IL1B −*31 T allele has been associated with increased IL1β secretion (36), and other haplotype TCT, for *IL1B −*511 C>T, *IL1B −*31 T>C, and *IL1B* +3954 C>T, in turn, has been considered a proinflammatory haplotype, with a predominant role of *ILB* *−*511 C>T (37). Moreover, association between cardiomyopathy and the *IL1B* +5810 G>A (rs1143633, intronic SNV) G allele and GG genotype was also identified, despite no established effect of this allele or genotype on IL1β production (35). It is possible, however, that other biological effects or linkage disequilibrium with other polymorphisms related to functional significance may exist. By comparing seronegative individuals with seropositive cardiac patients in Colombia, a study suggested protection against *T. cruzi* infection and development of cardiopathy for patients that carry the C allele of *IL17A* rs8193036 C>T (promoter SNV) (38). These data were *a priori* not expected, since the C allele has been associated with lower IL17A mRNA expression (39) and previous reports revealed a role of IL17A in resistance to *T. cruzi* in infected mice (27) and higher IL17A levels in patients with no cardiopathy (11, 12). It should be noted, however, that this study conducted with Colombian patients shows that the *IL17A* rs8193036 SNV is probably in linkage disequilibrium with some causative variant that modulates IL17A production (38). This investigation also found no association between the *IL17A* rs7747909 G>A 3’ UTR SNV and Chagas cardiopathy and between two other *IL17A* promoter SNVs (rs4711998 and rs3819024) or *IL17A* rs2275913 and heart disease, in a context where the latter A allele is associated with increased IL17 A levels (40, 41). A previous study, in turn, reported association of the *IL17A* rs2275913 G>A (promoter SNV) A allele and AA genotype with chronic cardiomyopathy in Brazil (42). This observation disagrees with previous studies that detected association between higher IL17A levels and absence of cardiomyopathy (11, 12) and are not in line with the often reported association between the A allele and increased IL17A levels (40, 41). Associations between *T. cruzi* infection and the *IL18* rs187238 C>A (promoter SNV) C, rs360719 A>G (promoter SNV) G, rs2043055 A>G (intronic SNV) A, and rs1946518 C>A (promoter SNV) A alleles were also found in Colombia, a finding mainly driven by the rs360719 G allele (43). Of note, this observation was confirmed in a larger cohort in Latin America (44). Importantly, another study detected association between the rs360719 G carrier status and higher serum IL18 mRNA expression in healthy individuals (45) while an additional report revealed increased IL18 mRNA expression at the myocardium level in Chagas disease cardiomyopathy (18). In addition, the *IL18* rs2043055 G allele was found to associate with absence of cardiomyopathy in Colombia (44). In Brazil the *IL18* rs2043055 AG genotype was more frequent in patients with a moderate than a severe cardiac phenotype. This study also suggested that it is possibly that this intronic SNV is in linkage disequilibrium with another variant functionally relevant (46). This SNV, however, has not yet been shown to affect IL18 production.

While meaningful progress has been made in linking cytokine gene polymorphisms to Chagas disease mechanisms, associations between cytokine-related SNVs with different clinical scenarios of this disease remain largely limited. Establishing their consequences in the corresponding cytokine circulating and local levels, moreover, as well as their implications on specific biological pathways at the host environment, comprise even more complexes problems to be addressed. In spite of such challenges, thoughtful and well supported hypotheses can be made to progressively add pieces to this ongoing puzzle. In this context, association studies involving appropriate cytokine-related SNVs and strategic immune-affected medical conditions, in one side, and key Chagas phenotypes, on the other side, should be encouraged as potentially significant contributors to the comprehension of this disease pathogenesis.

Facing the biological and clinical scenarios just described, in the current study we investigated potential associations between SNVs linked to *IL1B*, *IL6*, *IL17A*, or *IL18* and the risks of positive *T. cruzi* parasitemia or development/progression of cardiomyopathy. With the assumption that immunocompromised conditions frequently associated with Chagas disease could potentially bring key mechanistic and clinical insights to the understanding of this illness, we also analyzed possible associations involving HIV infection and Chagas disease phenotypes. Surprisingly, our data revealed that positiveness for HIV protected against the development and/or progression of cardiomyopathy. We also describe novel associations between specific *IL18*, *IL17A*, and *IL1B* variant profiles and the risk of cardiomyopathy as well as between a specific *IL6* polymorphic genotype and the risk of positive *T. cruzi* parasitemia.

## Patients, Materials, and Methods

### Study Subjects

Our cohort included 206 patients with Chagas disease: 78 from the Heart Institute of Hospital das Clínicas da Faculdade de Medicina, University of São Paulo (HCFMUSP), and 129 from the Infectious Diseases Division of HCFMUSP, 49 of whom with HIV co-infection. These 49 cases were followed at Serviço de Extensão e Atendimento aos Paciente com Infecção por HIV/Aids of the same Division. All participants were adults. The diagnosis of trypanosomiasis was based on the 2nd Brazilian Guidelines for Chagas Diseases (1). Two positive tests of the following ones, therefore, were used to establish the diagnosis: ELISA—enzyme linked immunosorbent assay, indirect immunofluorescence, and hemagglutination (47). All patients from the Infectious Diseases Division with positive epidemiology were sent to the HIV/Aids Clinic, where HIV infection was confirmed by ELISA and immunoblot (31). Patients from the Heart Institute were tested for HIV as previously described (31). When included in the current protocol, 26 of the 49 HIV patients were not on regular HAART therapy or had therapeutic failure due to antiretroviral resistance (three patients). In 23 of them, the median viral load was 14,000 (2,618–100,000, 25th–75th percentiles) DNA viral copies/µl while the median CD4 count was 340 (93–510) cells/mm^3^. Data from 23 patients under HAART for 1 to >5 years showed a median viral load of 0.0 (0.0–0.0) DNA viral copies/µl. In these 23 patients, the median CD4 count was 631 (439–715) cells/mm^3^.

The patients underwent electrocardiography, echocardiography, and thorax, esophagus, and colon radiological examinations, being then classified within one of the clinical forms of Chagas disease (1). Individuals without signs and symptoms and with no alteration in the mentioned tests were classified as having the indeterminate form (n = 51), patients with abnormalities suggestive of Chagas disease on electrocardiography or on dynamic electrocardiography and without digestive involvement as with the cardiac form (n = 96), the cases with abnormal findings on esophagus and/or colon without Chagas cardiac manifestation as presenting the digestive form (n = 32). Twenty-seven patients presented both digestive and cardiac alterations, constituting the cardio-digestive group. Cardiac involvement was detected in 123 patients (cardiac and cardiac/digestive forms) while heart disease was not identified in 83 patients, a group composed of individuals with the indeterminate and digestive forms.

Patients with cardiomyopathy were evaluated for heart failure according to the New York Heart Association (NYHA) classification (48), which includes the following criteria: class 1. Patients with cardiac disease without limitation of physical activity; class 2. Patients with cardiac disease with mild limitation of physical activity and symptomatic in routine physical activity; class 3. Patients with cardiac disease with marked limitation of physical activity and symptomatic in less than ordinary physical activity; and class 4. Patients with cardiac disease and unable to carry any physical activity without discomfort, symptoms may be present even at rest. For the purposes of our study, however, Chagas patients were classified according to two different scenarios related to their cardiac status, being divided in two groups in each of the cases: A) based on the NYHA classification/absence of cardiopathy—group 1: patients without cardiopathy (no CA) and with mild cardiopathy (NYHA 1), and group 2: patients with more severe cardiopathy (NYHA ≥ 2); and B) Based on the left ventricular ejection fraction (LVEF) yielded by echocardiography—group 1: patients with LVEF ≥ 45%, and group 2: patients with LVEF < 45%.

The control group for the SNV analyses consisted of 90 healthy individuals with negative serological tests for *T. cruzi* antigens. Controls and patients comprised a total population of 296 individuals, including 149 females and 147 males: 215 white and 73 non-white. This classification was not available for eight control individuals. Among the 206 patients included in this study, 135 were classified within these groups according to self-declaration; 71 of them, in turn, following a distinct institutional policy, were classified according to their government-issued identification document or by the hospital employee who processed their hospital registration.

### Ethics Statement

The present study was approved by the Ethics Committee for Research Project Analysis of Hospital das Clínicas da Faculdade de Medicina da Universidade de São Paulo (HCFMUSP), São Paulo, Brazil (CAPPesq 0174/11). All subjects gave written informed consent in accordance with the Declaration of Helsinki.

### Evaluation of Parasitemia

Patients’ peripheral blood samples were collected in EDTA (ethylenediaminetetraacetic acid) tubes. DNA extraction was performed using the QIAmp DNA Mini Kit (Qiagen). DNA concentration and purity were analyzed with a spectrophotometer. The parasitemia status was determined employing a qualitative polymerase chain reaction (PCR) performed with the S35/S36 primers designed to the kinetoplastic DNA of *T. cruzi* (Invitrogen, Thermo Fisher Scientific, Carlsbad, CA, USA), as previously described (49).

### DNA Extraction and Genotyping

In most patients genomic DNA was extracted from peripheral blood samples using the QIAampTM DNA Mini Kit (Qiagen, Hilden, Germany). For the cases the DNA concentration was inappropriately low, an additional extraction was carried out using the salt precipitation method (DTAB/CTAB; Sigma-Aldrich, Merck, St. Louis, MO, USA) (50). The DNA samples underwent spectrophotometric evaluation to assess yield and purity. SNVs were investigated by real-time PCR with the TaqMan^®^ Genotyping Master Mix assay and the corresponding SNV-specific primers in the StepOnePlus platform (Applied Biosystems, Thermo Fisher Scientific, Foster City, CA, USA). The investigated SNPs included *IL1B −*31 rs1143627 T>C, *IL6* *−*174 rs1800795 C>G, *IL17A* *−*152 rs2275913 G>A, *IL18* *−*137 rs187238 C>G, and *IL18* *−*607 rs1946518 C>A.

### Serum IL-18 Quantification

IL-18 levels were measured using commercial ELISA Kits (R&D Systems, Minneapolis, MN, USA) according to the manufacturer’s instructions. Sera from 43 patients, 15 HIV-infected and 28 non-infected, were stored at *−*70°C. Twenty-four of such patients had cardiac disease. At the time of blood collection, the median viral load in the 15 HIV-infected patients was 0.0 DNA copies/µl. In 14 of them the median of CD4 count was 873.5 (769.8–974.0) cells/mm^3^. Three of the 15 HIV-infected patients were not on regular antiretroviral therapy. Two had detectable viremia, with one of them displaying a CD4 count below 350 cells/mm^3^. The third is an HIV-1-infected patient with an undetectable viral load and high CD4 level despite the absence of HAART therapy, belonging to the rare group named as “elite suppressor” (51).

### Statistical Analyses

The calculations of Hardy-Weinberg equilibrium (HWE) were performed using chi square tests in MS Excel 365 (Microsoft Corporation, Redmond, WA). Multiple contingency analyses were performed to investigate potential associations among elected dependent variables and covariates. Fisher’s exact test was used for 2x2 contingency tables; otherwise the chi-square test was applied. When chi square test was used and was applicable, we performed a *post-hoc* test using standardized and adjusted residues with the Bonferroni correction. The P values obtained with single association tests (Fisher exact test or Pearson chi square test) were used as triage to elect candidate variables for multivariable analyses. We included in the logistic regression all variables that reached P < 0.2 in the single variable association test with the dependent variable. In the genetic models, when more than one model reached P < 0.2, we assumed the model with lower P value as the best-fit model and included it in the logistic regression analysis. To increase the modeling performance, continuous variables were categorized using a ROC (receiving operator characteristic) curve and clinical/biological criteria. Some variables presented missing values in 1–20% of cases and displayed a non-monotone missing pattern. In such cases, we performed multiple imputations including 100 imputed values for each missing one (52). This step was accomplished using MCMC (Markov chain-Monte Carlo simulation) for value imputation. The genotypes were clustered according to four possible models of genetic effects: additive (each genotype was analyzed separately), dominant (the presence of at least one SNV allele is associated with the effect), heterozygous (the heterozygous genotype is associated with the effect on phenotype), and recessive (the presence of SNV homozygosity is associated with the effect on phenotype). We adopted the best-fit model in the chi-square/Pearson exact test analyses, defined by the most significant P-value.

Since rs1946518 and rs187238 are just 470 bp apart from each other in the *IL18* promoter, we investigated linkage disequilibrium between these two SNVs using the Haploview software (53) and evaluated potential associations involving the haplotypes.

Chagas cardiopathy, NYHA (New York Heart Association) score, LVEF, and parasitemia were selected upfront as dependent variables for the multiple logistic regressions. Since there was potential interference of the clinical form in the analyzed endpoints, we considered the digestive form as an independent variable in addition to traditional potential confounding factors. To address this point, such a variable was included in the triage as a potential predictor in the model applied to the logistic regression analysis.

Continuous variables were tested for normality with the Shapiro-Wilk test. Continuous parametric variables were analyzed using *t* test to compare two groups, with the Welch correction if the Levine test pointed to unequal variance between the groups. Continuous non-parametric variables were analyzed using the Mann-Whitney U test when comparing two groups, or Kruskal-Wallis test when comparing more than two groups, followed by *post-hoc* analysis with the Bonferroni correction. Categorical variables were compared using the chi-square or Fisher exact test. Categorical variables are expressed as the number of cases and percentages while continuous variables as mean ± standard deviation when parametric, and median (25–75% range) when non-parametric. We accepted an α risk of 5% in this exploratory research. The statistical analyses were run in SPSS 24.

## Results

### Single Nucleotide Variant Genotype and Allele Distribution in the Analyzed Patient Population and Potential Implications

The studied Chagas disease patient population presented a balanced gender composition, predominance of whites and a median age of 50 (42-60) years (**Table 1**). Notably, 49 patients (23.8%) had HIV co-infection. The distribution of SNV-related genotypes, additional clinical features and the Brazilian state of birth are shown in **Supplementary Table 1**.

**Table 1:** Patient distribution according to demographic features, HIV status, parasitemia, cardiac phenotypes, and single nucleotide variant (SNV) genotypes.

Patient characteristics	n/median	%/25–75%
Age (years)	50.62	42.0–60.0
Age Range		
<35	23	11.2
35–50	88	42.7
>50	95	46.1
Skin color		
White	152	73.8
Non-white	54	26.2
Sex		
Female	103	50
Male	103	50
HIV		
HIV infection	49	23.8
No HIV infection	157	76.2
Parasitemia		
Positive	90	43.7
Negative	112	54.4
Missing	4	1.9
Chagas cardiopathy		
No	83	40.3
Yes	123	59.7
Clinical forms		
Indeterminate form	51	24.8
Cardiac form	96	46.6
Digestive form	32	15,5
Cardio-digestive form	27	13.1
NYHA		
NYHA < 2/no CA	79	38.3
NYHA ≥ 2	112	54.4
Missing	15	7.3
LVEF (%)	60	28–67
LVEF < 45%		
Negative	115	55.8
Positive	69	33.5
Missing	22	10.7
*IL1B* −31 rs1143627 T>C		
TT	68	33.0
TC	92	44.7
CC	46	22.3
*IL6* −174 rs1800795 C>G		
CC	12	5.8
CG	65	31.6
GG	129	62.6
*IL17A* −152 rs2275913 G>A		
GG	131	63.6
GA	62	30.1
AA	13	6.3
*IL18* −607 rs1946518 C>A		
CC	74	35.9
CA	93	45.1
AA	39	18.9
*IL18* −137 rs187238 C>G		
CC	111	53.9
CG	77	37.4
GG	18	8.7

HIV, human immunodeficiency virus; LVEF, left ventricular ejection fraction; NYHA, New York Heart Association score; no CA, without cardiopathy. n = 206 patients, except of IL18 levels (n = 43). When missing values were not shown those values are zero.

The entire study population was in Hardy-Weinberg equilibrium (HWE) (**Supplementary Table 2**). The genotype frequencies were also in HWE in whites and non-whites (**Supplementary Table 2**). Interestingly, HWE equilibrium was not present in some disease conditions. We detected HWE violation in *IL6* *−*174 rs1800795 C>G in patients with detectable *T*. *cruzi* DNA in peripheric blood; in patients without cardiopathy (*IL17* rs2275913 G>A and *IL18* −137 rs187238 C>G) and in patients with cardiopathy (*IL1B* rs1143627 T>C); in individuals with NYHA < 2 (NYHA 1) or without heart failure (*IL17* rs2275913 G>A and *IL18* *−*607 rs1946518 C>A); and in subjects with NYHA ≥ 2 (*IL1B* rs1143627 T>C) (**Supplementary Table 2**). Those data suggest that the mentioned disease conditions could be influenced by the allelic composition in the selected *loci*.

We analyzed self-declared/documented ancestry as a potential confounding factor for all dependent variables. The P values are mentioned in **Supplementary Table 3** and the comparisons can be appreciated in **Supplementary Table 4**, which comprises the frequencies of self-declared/documented ancestry for each of the dependent variables. The P values show that there was no significant interference of self-declared/documented ancestry in the analyzed comparisons carried out in this study.

### *IL1B, IL17A*, and *IL18* Polymorphic Genotypes Decrease the Risk or Attenuate Progression of Cardiomyopathy in Chagas Disease

Preliminary univariate analyses showed potential associations between cardiomyopathy and sex (P = 0.023), *IL18* *−*607 rs1946518 C>T applying the recessive model (P = 0.011), *IL18* *−*137 rs187238 C>G using the recessive model (P = 0.078), *IL17* rs2275913 G>A based on the recessive model (P = 0.039), and digestive involvement (P = 0.012) (**Supplementary Table 3**).

These initial analyses also predicted potential associations between NYHA score ≥ 2 and gender (*p* = 0.019), *IL1B* rs1143627 T>C using the heterozygous model (P = 0.008), *IL18* *−*607 rs1946518 C>A applying the recessive model (P = 0.009), *IL18* *−*137 rs187238 C>G applying the recessive model (P = 0.194), and digestive involvement (P = 0.001) (**Supplementary Table 3**). LVEF-based analyses, in turn, showed potential associations between LVEF < 45% and sex (P = 0.128), *IL18* −607 rs1946518 C>A applying the recessive model (P = 0.003), *IL18* *−*137 rs187238 C>G applying the recessive model (P = 0.052), *IL1B* rs1143627 T>C using the heterozygous model (P = 0.033), *IL6* rs 1800795 C>G applying the recessive model (P = 0.028), *IL17* rs2275913 G>A using the recessive model (P = 0.157), and digestive involvement (P < 0.001) (**Supplementary Table 3**).

As for Parasitemia, potential associations were shown with LVEF < 45% (P = 0.031), NYHA score ≥ 2 (P = 0.180), and *IL6* rs1800795 heterozygous model (P = 0.023) (**Supplementary Table 3**).

In this scenario, we performed multiple logistic regression analyses for each of the elected dependent variables. This assessment revealed that males with Chagas disease had an OR of 2.22 (P = 0.011, 95% CI 1.20–4.09) to develop cardiomyopathy (**Table 2**). Interestingly, homozygosity for the *IL17* rs2275913 A allele carried a heart protective effect, being associated with an OR of 0.27 (P = 0.044, 95% CI 0.07–0.97) (**Table 2**). A similar effect was detected for homozygosity of the *IL18* *−*607 rs1946518 A allele (OR = 0.35; P = 0.023, 95% CI 0.14–0.87).

**Table 2:** Multiple logistic regression for cardiopathy.

	OR	95% CI	P
Sex (male)	2.22	1.20–4.09	**0.011**
HIV	0.48	0.23–0.96	**0.039**
*IL17A* −152 rs2275913 G>A recessive	0.27	0.07–0.97	**0.044**
*IL18 −*607 rs1946518 C>A recessive	0.35	0.14–0.87	**0.023**
*IL18* −137 rs187238 C>G recessive	1.04	0.29–3.71	0.953
Digestive Involvement	0.55	0.28–1.05	0.069

95% CI, 95% confidence interval; HIV, human immunodeficiency virus; OR, odds ratio; P values ≤ 0.05 in bold.

Our analyses also established a positive association between NYHA score ≥ 2 and male gender (OR = 2.62, P = 0.007, 95% CI 1.30–5.27) and a negative association between this NYHA score range and the *IL18* *−*607 rs1946518 AA genotype (OR = 0.21, P = 0.009, 95% CI 0.06–0.68) and *IL1B* rs1143627 T>C using the heterozygous model (OR = 0.48, P = 0.036, 95% CI 0.24–0.95) and digestive involvement (OR = 0.20, P < 0.001, 95% CI 0.09–0.47) (**Table 3**). A similar effect was observed for the *IL18* *−*607 rs1946518 AA genotype with respect to LVEF < 45% (OR = 0.22, P = 0.034, CI 0.05–0.89), and digestive involvement (OR = 0.24, P = 0.004, 95% CI 0.09–0.62) (**Table 4**).

**Table 3:** Multiple logistic regression for New York Heart Association (NYHA) ≥ 2.

	OR	95% CI	P
Sex (male)	2.620	1.30–5.27	**0.007**
HIV	0.153	0.06–0.39	**<0.001**
*IL1B* −31 rs1143627 T>C heterozygous	0.476	0.24–0.95	**0.036**
*IL18* −607 rs1946518 C>A recessive	0.208	0.06–0.68	**0.009**
*IL18* −137 rs187238 C>G recessive	2.567	0.52–12.76	0.249
Parasitemia	0.801	0.39–1.63	0.539
Digestive Involvement	0.204	0.09–0.47	**<0.001**

95%, CI 95% confidence interval; HIV, human immunodeficiency virus; NYHA, New York Heart Association score; OR, odds ratio; P values ≤ 0.05 in bold.

**Table 4:** Multiple logistic regressions for left ventricular ejection fraction (LVEF) < 45%.

	OR	95% CI	P
Sex (male)	1.967	0.82–4.70	0.128
HIV	0.033	0.00–0.25	**0.001**
*IL1B* −31 rs1143627 T>C heterozygous	0.631	0.29–1.35	0.237
*IL6* −174 rs1800795 C>G recessive	13.082	0.40–431.30	0.149
*IL17A* −152 rs2275913 G>A recessive	0.578	0.05–7.41	0.673
*IL18* −607 rs1946518 C>A recessive	0.221	0.05–0.89	**0.034**
*IL18* −137 rs187238 C>G recessive	2.213	0.28–17.40	0.450
Parasitemia	0.581	0.26–1.30	0.186
Digestive involvement	0.235	0.09–0.62	**0.004**

95% CI, 95% confidence interval; HIV, human immunodeficiency virus; LVEF, left ventricular ejection fraction; OR, odds ratio; P values ≤ 0.05 in bold.

When investigating linkage disequilibrium between rs1946518 and rs187238, we found D` of 0.89 and r^2^ of 0.43, indicating strong linkage disequilibrium between the two *loci*. Our analysis also revealed that the haplotype *IL18* *−*607 C/*IL18* *−*137 C was associated with Chagas cardiopathy (P = 0.0186) and LVEF < 45% (P = 0.0149) (**Supplementary Table 5**). This same haplotype also reached a marginal P value for association with NYHA ≥ 2 (P = 0.0554). No haplotype involving these two SNVs showed significant association with the parasitemia status.

### A Specific *IL6* Polymorphism Profile Modulates the Risk of *Trypanosoma cruzi* Parasitemia

Based on univariate analyses, potential associations were predicted to occur between positive *T. cruzi* parasitemia and *IL6* rs1800795 C>G applying the heterozygous model (P = 0.023), NYHA ≥ 2 (P = 0.180) and HIV infection (P = 0.008) (**Supplementary Table 3**).

Notably, multiple logistic regression analyses uncovered a negative association between parasitemia and the *IL6* rs1800795 CG genotype (OR = 0.45, P = 0.015, 95% CI 0.24 *−*0.86) and a positive association between parasitemia and HIV infection (OR = 2.18, P = 0.039, 95% CI 1.04–4.58) (**Table 5**).

**Table 5:** Multiple logistic regressions for parasitemia.

	OR	95% CI	P
HIV	2.18	1.04–4.58	**0.039**
*IL6* −174 rs1800795 C>G heterozygous model	0.45	0.24–0.86	**0.015**
NYHA ≥ 2	1.06	0.43–2.58	0.900
LVEF < 45%	0.65	0.24–1.72	0.385

95% CI, 95% confidence interval; HIV, human immunodeficiency virus; LVEF, left ventricular ejection fraction; NYHA, New York Heart Association score; OR, odds ratio; P values ≤ 0.05 in bold.

### HIV Infection Is Protective Against Development and Progression of Cardiomyopathy in Chagas Disease

Our preliminary univariate analyses suggested a protective cardiac effect of HIV infection in patients with Chagas disease, based on the development of cardiomyopathy (P = 0.045), the NYHA score (P < 0.001), and the LVEF values (P < 0.001) (**Supplementary Table 3**).

To evaluate the potential effect of HIV infection on the cardiac form of Chagas disease, we initially compared the HIV population with the non-HIV patients with respect to potentially interfering parameters (**Table 6**). This analysis showed no differences in age, sex, groups (white x non white), and the SNV genotypes analyzed in the current study.

**Table 6:** Comparisons between Chagas disease patients with and without HIV infection.

	HIV n(%)/median (25–75%)	Non-HIV n(%)/median (25–75%)	P
Age^a^	49 (39–60.5)	50 (42–60)	0.379
Age range^b^			0.062
<35	10 (20.4)	13 (8.3)	
35-50	19 (38.8)	69 (43.9)	
>50	20 (40.8)	75 (47.8)	
Sex^c^			0.326
Male	28 (57.1)	75 (47.8)	
Female	21 (42.9)	82 (52.2)	
White^c^ Non-white	37 (75.5) 12 (24.5)	115(73.2) 42 (26.8)	0.853
Chagas cardiopathy^c^			**0.045**
No	26 (53.1)	57 (36.3)	
Yes	23 (46.9)	100 (63.7)	
NYHA ≥ 2^c^			**<0.001**
No			
Yes	8 (16.3)	71 (50.0)	
Missing	0	15	
Left ventricular ejection fraction^a^	0.67 (0.62–0.71)	0.43 (0.25–0.65)	**<0.001**
Ejection fraction ≥ 45%^c^	45 (97.8)	70 (50.7)	**<0.001**
Ejection fraction < 45%	1 (2.2)	68 (49.3)	
Missing	3	19	
Parasitemia: negative^c^	18 (38.3)	94 (60.6)	**0.008**
Parasitemia: positive	29 (61.7)	61 (39.4)	
Missing	2	2	
Clinical form^b^			**0.001**
Indeterminate form	20 (40.8)	31 (19.7)	
Cardiac form	11 (22.4)	85 (54.1)	
Digestive form	8 (16.3)	24 (15.3)	
Cardio-digestive form	10 (20.4)	17 (10.8)	
Genotypes			
*IL1B* −31 rs1143627 T>C^b^			0.168
TT	11 (22.4)	57 (36.3)	
TC	24 (49.0)	68 (43.3)	
CC	14 (28.6)	32 (20.4)	
*IL6* −174 rs1800795 C>G^b^			0.984
CC	3 (25.0)	9 (5.7)	
CG	15 (6.1)	50 (31.8)	
GG	31 (63.3)	98 (62.4)	
*IL17A −*152 rs2275913 G>A^b^			0.559
GG	33 (67.3)	98 (62.4)	
GA	12 (24.5)	50 (31.8)	
AA	4 (8.2)	9 (5.7)	
*IL18 −*607 rs1946518 C>A^b^			0.814
CC	16 (32.7)	58 (36.9)	
CA	24 (49.0)	69 (43.9)	
AA	9 (18.4)	30 (19.1)	
*IL18 −*137 rs187238 C>G^b^			0.180
CC	22 (44.9)	89 (56.7.)	
CG	20 (40.8)	57 (36.3)	
GG	7 (14.3)	11 (7.0)	

NYHA, New York Heart Association score. HIV patients, (n = 49), non-HIV patients, (n = 157). Comparisons: ^a^Student t test, ^b^Pearson chi square test, ^c^Fisher exact test. Percentual distributions considered only valid cases. When missing values were not shown those values are zero. P values ≤ 0.05 in bold.

Remarkably, multiple logistic regression analyses revealed a protective effect of HIV infection against the development of cardiomyopathy, displaying an OR of 0.48 (P = 0.039, 95% CI 0.23–0.96) (**Table 2**). In the same line, negative associations were shown for NYHA score ≥ 2 and HIV infection (OR = 0.15, P < 0.001, 95% CI 0.06–0.39) (**Table 3**) as well as for LVEF < 45% and positive HIV status (OR = 0.03, P < 0.001, 95% CI 0.00–0.25) (**Table 4**).

### IL18 Levels, Cardiopathy, New York Heart Association, Left Ventricular Ejection Fraction, and HIV

IL18 levels were analyzed in 43 patients and assessed with respect to the genotypes/alleles and the presence of cardiopathy, HIV infection, NYHA score, and LVEF (**Table 7**). The IL18 levels did not significantly vary among *IL18 −*607 or *IL18 −*137 SNV genotypes/alleles. No significant differences in this interleukin levels were detected either between patients with and without cardiopathy or HIV infection as well as among the different NYHA score or LVEF-related groups. Three patients without HAART therapy displayed serum levels of 737.49, 739.87, and 744.29 pg/ml. These last two were viremic and only one had a CD4 count below 350 cells/mm^3^. The 13 aviremic individuals under HAART therapy presented a median value of 308.24 pg/ml and all of them had CD4 cells higher than 350 cell/mm^3^.

**Table 7:** IL18 serum levels in Chagas disease patients according to the HIV status, cardiac phenotypes, parasitemia, and *IL 18 −*607 and *IL 18 −*137 genotypes.

	N	Serum level IL-18 (pg/ml)Median (25–75%)	P
HIV^a^			0.212
Negative	28	268.0 (190.3–736.0)	
Positive	15	736.6 (194.7–747.2)	
Cardiopathy^a^			0.903
No	22	301.3 (215.7–737.4)	
Yes	21	308.2 (176.4–753.4)	
NYHA^a^			0.508
NYHA <2/no CA	28	311.2 (215.0–753.2)	
NYHA ≥ 2	15	250.8 (176.7–739.5)	
LVEF^a^			0.782
LVEF ≥ 45%	28	298.3 (199.7–743.2)	
LVEF < 45%	14	284.9 (174.5–740.6)	
Missing	1		
Parasitemia^a^			0.131
Negative	20	247.2 (183.4–732.2)	
Positive	22	722.4 (214.209.7–751.5)	
Missing	1		
*IL18 −*607 rs1946518 C>A^b^			0.897
CC	17	314,1 (185.6–740.9)	
CA	16	313.7 (221.0–742.3)	
AA	10	275.2 (182.4–741.8)	
*IL18 −*607 rs1946518 C>A^a^			0.944
CC+CA	33	275.2 (182.4–741.8)	
AA	10	314.2 (204.7–740.8)	
*IL18 −*137 rs187238 C>G^b^			0.878
CC	24	316.6 (185.3–742.6)	
CG	15	308.2 (216.1–744.0)	
GG	4	275.2 (206.6–623.8)	
*IL18 −*137 rs187238 C>G^a^			0.702
CC+CG	39	314.2 (194.7–744.0)	
GG	4	275.2 (206.6–623.8)	

HIV, human immunodeficiency virus; LVEF, left ventricular ejection fraction; NYHA, New York Heart Association score; no CA, without cardiopathy. Tests: ^a^Mann Whitney U test, ^b^Kruskal Wallis test. Total number of patients = 43. Percentual distributions considered only valid cases. When missing values were not shown those values are zero.

## Discussion

### *IL6, IL1B, IL17A*, and *IL18* Single Nucleotide Variants and Parasitemia in Chagas Disease

In a still largely unclear scenario relating cytokines and pathogenesis of Chagas disease, our study seeked for potential associations between strategic cytokine-related SNVs and key Chagas phenotypes restricted to the disease or combined with immune dysregulation.

*T. cruzi* parasitemia has been recognized as an element that accelerates chronic Chagas cardiopathy (19, 20, 22), although some reports question this effect (23–25). PCR-based *T. cruzi* detection in human biopsies supports that parasite persistence in the heart favors the progression of cardiomyopathy (19, 21). Identification of *T. cruzi* DNA in human blood, moreover, has been associated with a higher risk of development and deterioration of cardiomyopathy (20, 22).

IL6 is a key cytokine in mouse-induced *T. cruzi* myocarditis, triggering protection mechanisms in cardiomyocytes but favoring a higher parasite load (29). Acute *T. cruzi* experimental infection in mice with high inoculum, in fact, was followed by increased IL-6, high parasite load in tissue and marked inflammation (54, 55) while *IL6*-KO mice developed different levels of parasite load in the blood and myocardium depending on the *T. cruzi* strain (29, 54). These animals, however, presented increased mortality (29, 54). Such findings suggest that avoidance of high IL6 levels may play a role in lowering the parasite load whereas a certain tissue level of IL6 must be reached to allow a protective response against the parasite-induced lesion. A balanced level of IL6, therefore, is expected to reduce tissue damage and cardiomyocyte death, mitigating the disease. Of note, our data revealed that *IL6 −*174 CG heterozygosity is protective against *T. cruzi* parasitemia. This original finding raises an enticing mechanistic hypothesis for the relationship between IL6 and parasitemia. This SNV is located in the *IL6* promoter, having the G allele been associated with higher IL6 blood levels than the C allele (33, 34, 56, 57). It is likely, therefore, that the low IL6 levels associated with the CC genotype lead to a lack of immune-mediated protection against parasitemia, while the high IL6 levels observed in GG individuals create a relatively severe inflammatory background that also favors *T. cruzi* replication. On the other hand, a balanced scenario of protection and controlled inflammation, displayed by heterozygous patients, would provide the best setting to fight parasitemia.

Facing the possible role of IL18 in reducing parasitic load in *IL18*-KO mice (26), we investigated the influence of *IL18* SNVs on *T. cruzi* parasitemia. The *IL18* human gene harbors several SNVs in its promoter. *IL18* *−*607 C>A and *IL18* *−*137 C>G are located in nuclear factor binding sites for CREB (cyclic AMP-responsive element binding protein) and H4TF-1 (human histone H4 gene specific transcription fator-1), respectively. Variants of these two sites can not only affect *IL18* transcription but also IFNγ expression (58). The *−*607 A allele and AA genotype have been associated with lower *IL18* transcriptional rate and/or protein levels than the C allele and CC genotype in different clinical and physiological settings (59–63), whereas the haplotype C-607/G-137 is associated with higher expression of IL18. Our analyses, however, did not identify a significant impact of *IL18* *−*607 or *IL18* *−*137 alleles/genotypes on the *T. cruzi* parasitemic status.

The observation of increased parasitemia in *IL17A*-KO mice infected by *Leishmania infantum* supports a role of IL17A in controlling parasitemia (64). IL17A and IFNγ activate macrophages to phagocyte *T. cruzi* for further killing in the endosomal/lysosomal compartment (65). This finding is not a surprise, since association between higher levels of IL17A and resistance to *Leishmania donovani* had been previously reported (66).

The *IL17A* *−*152 G>A SNV is a critical regulator of the *IL17A* promoter, being located within a binding motif for NFAT (nuclear factor activated T cells) (67). The A allele is more often associated with higher IL17A serum levels (40, 41, 67). Despite these observations, our analysis did not show association between *IL17A* *−*152 G>A and *T. cruzi* parasitemia, not supporting a protective role of the A allele against parasitemia in human Chagas disease.

### Effects of *IL1B, IL17A, IL18, and IL6* Single Nucleotide Variants on Cardiomyopathy in Chagas Disease

Previous studies showed direct correlation between IL-6 serum levels and cardiomyopathy (7–9) and an inverse correlation between IL-6 levels and LVEF in the setting of cardiopathy (7). Based on these observations and the association of *IL6* −174 C with lower IL6 levels (33, 34, 56, 57), we hypothesized that this allele might be protective. Our findings, however, did not reveal such an impact, corroborating results from Colombia and Peru obtained in an ethnically distinct population affected by different parasite lineages (32, 68). It is possible, therefore, that other factors may also play a role in determining IL6 levels that modulate the cardiac phenotype in Chagas disease.

IL1β triggers an inflammation cascade, leads to generation of reactive free radicals, and may promote IFNγ production (69) and facilitate the differentiation of pro-inflammatory cells such as human Th17 (70). The assessed *IL1B* **−**31 T>C transition causes disruption of a TATA‐box, potentially changing the binding site affinity to regulatory proteins (36). We showed association between the *IL1B −*31 TC genotype and lower NYHA in Chagas disease. It is known that IL1β promotes inflammatory activity in acute experimental myocarditis induced by *T. cruzi* (71) and induces hypertrophy in primary cultures of cardiomyocytes infected by this parasite (72). Based on these data and because the *IL1B* −31 T allele is associated with increased *IL1B* transcription (36), heterozygosity may attenuate inflammation and cardiomyocyte hypertrophy in Chagas myocarditis, still allowing enough IL1β to fight *T. cruzi* infection. This balanced response likely explains the observed protective effect of the *IL1B −*31 TC genotype against cardiac disease severity. Interestingly, the CT haplotype for *IL1B −*31 T>C and +3,954 C>T was more prevalent in patients with Chagas cardiomyopathy in a Colombian population (35).

Higher frequencies of CD4+ IL-17A+ T-cells, lower levels of IL10 and IL10, and higher levels of IFNγ and TNFα are observed in individuals with more severe cardiomyopathy (6, 7). These findings are in agreement with the observation of lower lymphocyte IL17A expression in chronic Chagas cardiomyopathy than in seronegative individuals and patients with the indeterminate form (6). In addition, high IL17A levels were also associated with better heart function in Chagas disease (11). Based on these observations, IL17A was expected to potentially modulate inflammation in cardiac tissue and protect against *T. cruzi* infection (27, 28). A study in Brazil, however, unexpectedly revealed associations between the *IL17A* −152 A allele and the AA genotype with chronic cardiomyopathy (42). In contrast, another study in a Colombian cohort reported a protective effect of the C Allele of *IL17A* rs 8193036 C>T in seronegative individuals in comparison with cardiac seropositive patients (38), in a context of previously established association between the C allele and lower IL 17A mRNA expression (39). These data were *a priori* not expected, since previous reports showed a role for IL17A in mouse resistance to *T. cruzi* infection (27) and/or higher IL17A levels in patients without cardiopathy (11, 12). This study also found no association between the *IL17A* G>A 3’ UTR rs7747909 SNV, other two *IL17A* promoter SNVs (rs4711998 and rs3819024) or *IL17A* rs2275913 and Chagas cardiopathy.

In agreement with the expected role of IL17A, but in contrast to most clinical results, we found association between *IL17A* rs2275913 G>A and cardiac protection following a recessive model. Indeed, carriers of the A allele and the AA genotype were found to have a lower risk to develop cardiomyopathy. Since A allele has been found in association with higher IL17A serum levels (40, 41, 67), our data are in accordance with reports that show association between better cardiac function and higher IL17 A levels (11, 12). Our findings suggest that IL17A modulates the host inflammatory response to parasites in the myocardium, impacting the risk of developing cardiac dysfunction. Additional studies are necessary, however, to establish the effect of the AA genotype on IL17A serum levels in Chagas disease patients. It must be pointed out that our study differs from the previous ones (38, 42) with respect to patient geographical origin, ethnicity, classification, sample size, subgroup design, and possibly parasite lineage (68). Given the significant ethnical differences among Brazilian, Colombian, and Bolivian populations, genetic background may be a major determinant for the different results.

Since IL18 induces pathological cardiac remodeling (16), cardiomyocyte hypertrophy (73) and increased fibronectin expression (74), high levels of IL18 are expected to lead to cardiac hypertrophy and fibrosis, with a deleterious impact on heart function. IL18 is expressed in myocardium early in the course of experimental myocarditis induced by *T. cruzi* (15), leading to production of IFNγ. IL18 upregulation and correlation between IL18 mRNA and IFNγ expression have been shown in human Chagas heart tissue (18). Standing as a novel observation, we showed association between the *IL18 −*607 AA genotype and decreased risk of developing cardiomyopathy, progressing to NYHA ≥ 2, and progressing to LVEF < 45%. These data are in accordance to previously reported association between this AA genotype and low IL18 production in the presence and in the absence of mitogen stimuli (60–66). Unlike these studies, however, our data did not reveal significant associations between IL18 levels and any of the *IL18 −*607 or *IL18* −137 genotypes/alleles. Such differences may rely on the nature and size of the studied populations (55–101 patients), including other diseases, and/or the nature the biological samples and/or experimental conditions, including plasma and mucosal biopsies and peripheral blood cells under mitogen stimuli (58–63). The lack of association between IL18 serum levels and *IL18 −*607 C>A or *IL18* −137 C>G genotypes/alleles observed in our study does not allow a straight association between the mentioned cardiac findings and IL18 serum levels. This finding raises the hypothesis that IL18 serum levels may not significantly correlate with IL18 heart tissue levels in Chagas disease. However, since our study did not reproduce the association between IL18 levels and the *IL18* −607 SNV in other contexts, more representative patient samples should be evaluated under different conditions to more conclusively investigate this relationship in this disease. It must be noted that a previous study conducted in Colombian and Latin American Chagas patients did not detect significant frequency differences of *IL18 −*607 A allele and AA genotype between asymptomatic patients and ones with cardiac dysfunction (43, 44). Data from an Argentinian population, on the other hand, display a trend similar to our study (44). Our data strengthens the potential benefit of targeting IL18 as a therapy in Chagas cardiomyopathy. IL18 levels have indeed been associated with increased mortality and are inversely correlated with LVEF in heart failure (75). Moreover, neutralization of IL18 has been proposed in heart failure (76), Fabry cardiomyopathy (77) and sepsis-induced cardiac dysfunction (78). Our data also revealed association of the *IL18* −607 C/*IL18* −137 C haplotype with Chagas cardiopathy and LVEF < 45%. Our results on *IL18* −137, however, do not support an additive susceptibility status linking such SNVs.

Our study revealed that men have higher risks for cardiopathy and NYHA ≥ 2. These findings are in line with previous data that report male gender as one of the six independent variables that constitute risk factors for death in Chagas heart disease (3, 79). Male sex, therefore, appears as a robust risk factor for worse prognosis in Chagas cardiomyopathy. As a novel finding, digestive involvement associated negatively with NYHA ≥ 2 and LVEF < 45%, suggesting that such patients may develop a milder cardiopathy than affected individuals without digestive disease. This observation has never been reported (1, 31, 80). Alternative explanations for this finding may comprise a potential inclusion of more severely affected cardiac patients in our study and a potentially smaller than real proportion of patients with the indeterminate form due to underdiagnosis and/or loss of follow-up, raising the cardiac form/indeterminate form ratio. The relatively high proportion of cardio-digestive cases previously reported (1, 31), however, adds further support to the influence of digestive involvement in NYHA and LVEF.

### Impact of HIV Infection on Development and Progression of Cardiomyopathy in Chagas Disease

In immunosuppressed patients with HIV infection, elevated IL18 serum concentrations have been shown to favor the development of Aids and associated infections (30). In addition, Chagas disease reactivation does not occur in immunocompetent subjects, while in HIV-infected patients this illness has been associated with increased morbidity and mortality (31). Based on a severe combined immunodeficient (SCID) animal model (81), this observation may be explained by dissociation between a high parasite load and lower tissue inflammation, which is associated with decreased IFNγ levels and early mortality. Interestingly, *T. cruzi* inhibits HIV replication in macrophage-derived monocyte culture (82). In our study the HIV infected and non-infected groups did not differ with respect to the evaluated SNV profiles, age or gender. In the HIV-infected group, 53.1% of the patients were not on HAART therapy. Our findings revealed higher parasitemia in HIV-infected Chagas individuals, confirming previous data (83). Of note, in *T. cruzi*/HIV coinfection a Th2 response likely contributes to the impairment of parasite control (84). Altogether, our data suggest that antiretroviral therapy interferes positively in the host-HIV-*T. cruzi* interaction.

To our surprise, our analyses revealed a previously not described finding: HIV infection exerts a strong protective effect on Chagas disease cardiomyopathy. HIV infection was associated with lower risks to develop cardiopathy and to progress to NYHA ≥ 2 and LVEF < 45%. This *a priori* unexpected effect may be based on actions of the HAART regimens, reported to decrease the prevalence of HIV-associated cardiomyopathy in about 30% of the HIV-infected patients (85). In contrast, HAART failure was associated with continued IL18 elevation (86).

IL18 contributes to Aids pathogenesis by increasing viral replication, death of endothelial cells, and cardiomyocyte proliferation (30). A previous study from our service showed that Chagas reactivation occurred only in patients not in HAART therapy, and progression to a more severe chronic disease and death were observed in 35 and 30% of them, respectively (31). Moreover, progression to severe chronic forms was verified in less than 10% of patients on HAART. In those under HAART therapy, we detected high levels of IL18 (>700 pg/ml) in two of viremic cases and a median of 308.24 pg/ml in 13 aviremic individuals. The small number of analyzed patients, however, did not allow statistical evaluation with respect to IL18 levels and viral load, and no differences in IL18 levels were detected between HIV and non-HIV patients. A previous report did not find difference between 32 HIV viremic and 15 aviremic patients either (87). While our data suggest that IL18 serum levels may not correlate with IL18 levels in cardiac tissue in Chagas disease, a larger future study should address a still possible IL18-reducing effect of HAART on Chagas cardiomyopathy.

Another potential explanation for this positive impact of HIV infection on Chagas disease could be a growth-reduction effect of antiretroviral drugs on *T. cruzi*. HIV protease inhibitors have in fact been reported to restrain epimastigote, trypomastigote, and amastigote growth (88–90). Pretreatment of trypomastigotes with nelfinavir and lopinavir inhibited their association with LLC-MK_2_ epithelial cells and RAW macrophages and decreased the number of intracellular amastigotes (88). In addition, interaction of ritonavir, lopinavir and nelfinavir and *T. cruzi* epimastigotes showed potent anti-proliferative effects and led to changes in differentiation (89).

Nelfinavir and lopinavir target vital cellular structures of trypomastigotes, leading to irreversible, lethal metabolic injuries in the size and structure (90). A trypanocidal effect of HIV proteinase inhibitors on the *T. cruzi* aspartyl peptidase intracellular target was also reported using a docking simulation model (91), showing a great affinity of ritonavir and lopinavir to bind to this active enzyme intracellular site. In this setting, saquinavir has been integrated to the computer-guided drug repositioning as a potentially new treatment for Chagas disease (92).

A synergistic interaction between a Th2 predominant response in *T. cruzi-*HIV infected patients and HAART could also potentially lead to reduction in IL18 levels (30). This combined effect might decrease the predominant Th1 response in myocardium (5) and reduce inflammatory cardiac lesions. Other cardiovascular actions of antiretrovirals are unlikely to contribute to the cardioprotective effect, since HAART has been linked to increased risk of cardiovascular illness (30).

While the scientific relevance and possible clinical applications raised by the HIV-related cardioprotective effect on Chagas cardiomyopathy are potentially remarkable, its underlying mechanisms remain unclear. Future studies with larger and more diverse coinfected patient populations, expansion of cardiac function/lesion markers, detailed characterization of the cardiac microenvironment associated this coinfection, and deep characterization of cardiac tissue response to HAART should bring significant contributions to this elucidation.

Our study brought novel and meaningful data to field of Chagas disease, but it also carries some limitations. Such limitations comprise the inclusion of a single center, Brazilian heterogeneous ethnicity, limited analyses of interleukin levels and respective functional impact, and lack of data integration involving the use of HAART, CD4+ count, HIV load, and molecular genetics assessment. The appropriate statistical model employed for small samples and the level of significance detected in key analyses, however, strongly support our conclusions and provide a robust platform for sequential future studies.

In conclusion, our findings revealed a novel association between the *IL6* −174 C>G SNV and parasitemia in Chagas disease, expressed as a decreased risk of its detection in patients with the CG genotype. As additional original findings, our data showed that the *IL17A −*152 AA and the *IL18* −607 AA genotypes are associated with a reduced risk of developing cardiomyopathy in individuals affected by this illness. The *IL18* −607 AA and *IL1B* TC genotypes were also found to associate with a lower risk of manifesting severe cardiac dysfunction. Most of our findings are consistent with previously reported clinical data on Chagas disease and data on cytokine expression. Unexpectedly, our results uncovered a novel protective effect of HIV infection in Chagas disease patients, translated into a decreased risk of developing cardiomyopathy. Available information suggests that a synergistic effect of HIV and HAART may mitigate a Th1-mediated response in the myocardium. The findings revealed by this report not only expand the understanding of Chagas disease pathogenesis and its relationship with other clinical scenarios but also add pieces of information to the field that can be potentially employed in the development of therapeutic interventions.

## Data Availability Statement

The datasets generated for this study can be found in the DRYAD Digital Repository (doi: 10.5061/dryad.wstqjq2hn).

## Author Contributions

MS-Y, VF, LO: conception and design. AG, EW, DF, JO, EN, CO, EB, AP, CN, FC, NC, PS, EY-K, VF, LO, MS-Y: analysis and interpretation. AG, EW, LO, MS-Y: drafting the manuscript for important intellectual content. All authors contributed to the article and approved the submitted version.

## Funding

This work was supported by São Paulo Research Foundation (FAPESP) [#12/50273-0]; and by Conselho Nacional de Desenvolvimento Científico e Tecnológico - Programa Institucional de Bolsas de Iniciação Científica (CNPq - PIBIC) [AGS #100793/2016-9 from January to July 2016], and JOR # 157966/2015-1 from September to December 2015]; and by FAPESP (Technical Training Scholarship [CSO # 2014/07100-3 from May of 2014 to March 2015] and Secretary of Administration of São Paulo State Secretary [DTF # from March of 2014 to February of 2016], and for IL18 analyses by São Paulo Research Foundation (FAPESP) [#19/06363-4].

## Conflict of Interest

The authors declare that the research was conducted in the absence of any commercial or financial relationships that could be constructed as a potential conflict of interest.

The reviewers FD and CM-J declared a shared affiliation, with no collaboration, with the authors to the handling editor at the time of the review.
