# Supplementary material for: A Specific IL6 Polymorphic Genotype Modulates the Risk of Trypanosoma cruzi Parasitemia While IL18, IL17A, and IL1B Variant Profiles and HIV Infection Protect Against Cardiomyopathy in Chagas Disease
Source: Front Immunol. 2020 Oct 22;11:521409. doi: 10.3389/fimmu.2020.521409 (PMC7642879; doi:10.3389/fimmu.2020.521409)
Supplement: Supplementary file 1 [file Table_1.pdf]

Supplementary Table 1 – Distribution of patients according to the sociodemographic characteristics, clinic of origin and genotypes

**Patient Characteristics**	**n**	**%**
State of birth		
Acre	1	0.5
Alagoas	5	2.4
Bahia	48	23.3
Bolivia (non-brazilian)	1	0.5
Ceará	4	1.9
Goiás	1	0.5
Manhão	1	0.5
Minas Gerais	50	24.3
Mato Grosso do Sul	1	0.5
Mato Grosso	1	0.5
Pará	1	0.5
Paraíba	4	1.9
Pernambuco	10	4.9
Piauí	5	2.4
Paraná	10	4.9
Rio Grande do Sul	1	0.5
Santa Catarina	1	0.5
São Paulo	60	29.1
Missing values	1	0.5
Total	206	100.0
Region of birth		
North	2	1.0
North East	76	36.9
Mid West	3	1.5
South East	110	53.4
South	12	5.8
Total	203	98.5
Missing Values	3	1.5
Clinic of origin		
HIV ambulatory	49	23.8
Infectious Disease	82	39.8
Heart Institute	75	36.4

Total	206	100.0
IL1B rs -31 1143627 T>C		
TT	68	33.0
TC	92	44.7
CC	46	22.3
Total	206	100.0
IL1B -31 rs1143627 Dominant		
TT+TC	138	67.0
CC	68	33.0
Total	206	100.0
IL1B -31 rs1143627 Heterozigous		
TT+CC	114	55.3
TC	92	44.7
Total	206	100.0
IL1B -31 rs1143627 Recessive		
TC+CC	160	77.7
TT	46	22.3
Total	206	100.0
IL6 -174 rs1800795 C>G		
CC	12	5.8
GC	65	31.6
GG	129	62.6
Total	206	100.0
IL6 -174 rs1800795 Dominant		
CC+CG	77	37.4
GG	129	62.6
Total	206	100.0
IL6 -174 rs1800795 Heterozigous		
CC+GG	141	68.4
CG	65	31.6
Total	206	100.0
IL6 -174 rs1800795 Recessive		
CC	12	5.8
GG+CG	194	94.2
Total	206	100.0
IL17A -152 rs2275913 G>A		
GG	131	63.6

GA	62	30.1
AA	13	6.3
Total	206	100.0
IL17A -152 rs2275913 Dominant		
GG	131	63.6
GA+AA	75	36.4
Total	206	100.0
IL17A rs2275913 -152 Heterozigous		
GG+AA	144	69.9
GA	62	30.1
Total	206	100.0
IL17A rs2275913 -152 Recessive		
GG+GA	193	93.7
AA	13	6.3
Total	206	100.0
IL18 -607 rs1946518 C>A		
CC	74	35.9
CA	93	45.1
AA	39	18.9
Total	206	100.0
IL18 -607 rs1946518 Dominant		
AA+CA	132	64.1
CC	74	35.9
Total	206	100.0
IL18 -607 rs1946518 Heterozigous		
AA+CC	113	54.9
CA	93	45.1
Total	206	100.0
IL18 -607 rs1946518 Recessive		
AA	39	18.9
CA+CC	167	81.1
Total	206	100.0
IL18 -137 rs187238 C>G		
CC	111	53.9
CG	77	37.4
GG	18	8.7
Total	206	100.0

IL18 -137 rs187238 Dominant		
CC	111	53.9
GG+CG	95	46.1
Total	206	100.0
IL18 -137 rs187238 Heterozigous		
CC+GG	129	62.6
CG	77	37.4
Total	206	100.0
IL18 -137 rs187238 Recessive		
CC+CG	188	91.3
GG	18	8.7
Total	206	100.0

HIV - Human immunodeficiency virus
