# Supplementary material for: A Specific IL6 Polymorphic Genotype Modulates the Risk of Trypanosoma cruzi Parasitemia While IL18, IL17A, and IL1B Variant Profiles and HIV Infection Protect Against Cardiomyopathy in Chagas Disease
Source: Front Immunol. 2020 Oct 22;11:521409. doi: 10.3389/fimmu.2020.521409 (PMC7642879; doi:10.3389/fimmu.2020.521409)
Supplement: Supplementary file 2 [file Table_2.pdf]

Supplemmentary Table 2. Analysis of Hardy-Weinberg Equilibrium in the patient population and subgroups

***IL1B* -31 rs1143627 T>C**							
		%		%		%	P
Genotype	TT		TC		CC		
All patients	68	33.0	92	44.7	46	22.3	0.166
Non-white	16	29.6	24	44.4	14	25.9	0.419
White	52	34.2	68	44.7	32	21.1	0.270
HIV negative	57	36.3	68	43.3	32	20.4	0.163
HIV positive	11	22.4	24	49.0	14	28.6	0.907
Cardiopathy: no	25	30.1	41	49.4	17	20.5	0.980
Cardiopathy: yes	43	35.0	51	41.5	29	23.6	0.076
NYHA <2/No CA	35	31.3	56	50.0	21	18.8	0.867
NYHA ≥ 2	33	41.8	24	30.4	22	27.8	**0.001**
Missing	0		12		3		
LVEF≥45%	34	29.6	59	51.3	22	19.1	0.689
LVEF<45%	27	39.1	24	34.8	18	26.1	**0.015**
Missing	7		9		6		
Parasitemia: no	40	35.7	46	41.1	26	23.2	0.080
Parasitemia: yes	26	28.9	45	50.0	19	21.1	0.954
Missing	2		1		1		

***IL6* -174 rs1800795 C>G**							
		%		%		%	P
Genotype	CC		CG		GG		
All patients	12	5.8	65	31.6	129	62.6	0.326
Non-white	3	5.6	13	24.1	38	70.4	0.212
White	9	5.9	52	34.2	91	59.9	0.667
HIV negative	9	5.7	50	31.8	98	62.4	0.441
HIV positive	3	6.1	15	30.6	31	63.3	0.525
Cardiopathy: no	3	3.6	28	33.7	52	62.7	0.745
Cardiopathy: yes	9	7.3	37	30.1	77	62.6	0.139
NYHA <2/No CA	5	4.5	36	32.1	71	63.4	0.873
NYHA ≥ 2	7	8.9	26	32.9	46	58.2	0.249
Missing	0		3		12		
LVEF≥45%	2	1.7	36	31.3	77	67.0	0.337
LVEF<45%	7	10.1	22	31.9	40	58.0	0.150
Missing	3		7		12		
Parasitemia: no	4	3.6	44	39.3	64	57.1	0.281
Parasitemia: yes	8	8.9	21	23.3	61	67.8	**0.007**

Missing	0		0		4		

***IL17A* -152 rs2275913 G>A**

		%		%		%	P
Genotype	GG		GA		AA		
All patients	131	63.6	62	30.1	13	6.3	0.135
Non-white	36	66.7	15	27.8	3	5.6	0.405
White	95	62.5	47	30.9	10	6.6	0.217
HIV negative	98	62.4	50	31.8	9	5.7	0.441
HIV positive	33	67.3	12	24.5	4	8.2	0.085
Cardiopathy: no	52	62.7	22	26.5	9	10.8	**0.012**
Cardiopathy: yes	79	64.2	40	32.5	4	3.3	0.695
NYHA <2/No CA	71	63.4	31	27.7	10	8.9	**0.024**
NYHA ≥ 2	50	63.3	26	32.9	3	3.8	0.867
Missing	10		5		0		
LVEF≥45%	76	66.1	31	27.0	8	7.0	0.067
LVEF<45%	43	62.3	25	36.2	1	1.4	0.209
Missing	12		6		4		
Parasitemia: no	71	63.4	33	29.5	8	7.1	0.144
Parasitemia: yes	57	63.3	28	31.1	5	5.6	0.531
Missing	3		1		0		

***IL18* -607 rs1946518 C>A**

		%		%		%	P
Genotype	CC		CA		AA		
All patients	74	35.9	93	45.1	39	18.9	0.313
Non-white	20	37.0	23	42.6	11	20.4	0.363
White	54	35.5	70	46.1	28	18.4	0.528
HIV negative	58	36.9	69	43.9	30	19.1	0.248
HIV positive	16	32.7	24	49.0	9	18.4	1.000
Cardiopathy: no	26	31.3	34	41.0	23	27.7	0.102
Cardiopathy: yes	48	39.0	59	48.0	16	13.0	0.748
NYHA <2/No CA	39	34.8	44	39.3	29	25.9	**0.028**
NYHA ≥ 2	30	38.0	41	51.9	8	10.1	0.266
Missing	5		8		2		
LVEF≥45%	36	31.3	51	44.3	28	24.3	0.244
LVEF<45%	29	42.0	35	50.7	5	7.2	0.200
Missing	9		7		6		
Parasitemia: no	38	33.9	54	48.2	20	17.9	0.914
Parasitemia: yes	35	38.9	36	40.0	19	21.1	0.099
Missing	1		3		0		

***IL18* -137 rs187238 C>G**							
		%		%		%	P
Genotype	CC		CG		GG		
All patients	111	53.9	77	37.4	18	8.7	0.381
Non-white	33	61.1	20	37.0	1	1.9	0.298
White	78	51.3	57	37.5	17	11.2	0.191
HIV negative	89	56.7	57	36.3	11	7.0	0.653
HIV positive	22	44.9	20	40.8	7	14.3	0.487
Cardiopathy: no	43	51.8	29	34.9	11	13.3	0.103
Cardiopathy: yes	68	55.3	48	39.0	7	5.7	0.697
NYHA <2/No CA	60	53.6	40	35.7	12	10.7	0.186
NYHA ≥ 2	43	54.4	32	40.5	4	5.1	0.527
Missing	8		5		2		
LVEF≥45%	59	51.3	43	37.4	13	11.3	0.239
LVEF<45%	40	58.0	27	39.1	2	2.9	0.306
Missing	12		7		3		
Parasitemia: no	61	54.5	44	39.3	7	6.3	0.802
Parasitemia: yes	48	53.3	31	34.4	11	12.2	0.105
Missing	2		2		0		

No CA: without cardiopathy, LVEF: left ventricular ejection fraction. NYHA: New York Heart Association score. Percentual distributions considered only valid cases. When missing values were not shown those values are zero. P values ≤ 0.05 in bold.
