# Supplementary material for: A Specific IL6 Polymorphic Genotype Modulates the Risk of Trypanosoma cruzi Parasitemia While IL18, IL17A, and IL1B Variant Profiles and HIV Infection Protect Against Cardiomyopathy in Chagas Disease
Source: Front Immunol. 2020 Oct 22;11:521409. doi: 10.3389/fimmu.2020.521409 (PMC7642879; doi:10.3389/fimmu.2020.521409)
Supplement: Supplementary file 3 [file Table_3.pdf]

Supplementary Table 3. Multiple chi square/Fisher exact tests for elected enpoints

**NYHA ≥2**	P
NYHA >2 vs White/Non-white[a]	1.000
NYHA ≥2 vs Sex[a]	**0.019**
NYHA ≥2 vs HIV[a]	**< 0.001**
NYHA ≥2 vs *IL1B* -31 rs1143627 T>C Genotypes[b]	**0.025**
NYHA ≥2 vs *IL1B* -31 rs1143627 Dominant model[a]	0.161
NYHA ≥2 vs *IL1B* -31 rs1143627 Heterozygous[a]	**0.008**
NYHA ≥2 vs *IL1B* -31 rs1143627 Recessive model[a]	0.167
NYHA ≥2 vs *IL6* -174 rs1800795 C>G Genotypes[b]	0.441
NYHA ≥2 vs *IL6* -174 rs1800795 Dominant model[a]	0.547
NYHA ≥2 vs *IL6* -174 rs1800795 Heterozygous model[a]	>0.999
NYHA ≥2 vs *IL6* -174 rs1800795 Recessive model[a]	0.239
NYHA ≥2 vs *IL17A* -152 rs2275913 G>A Genotypes[b]	0.330
NYHA ≥2 vs *IL17A* -152 rs2275913 Dominant model[a]	1.000
NYHA ≥2 vs *IL17A* -152 rs2275913 Heterozygous model[a]	0.521
NYHA ≥2 vs *IL17A* -152 rs2275913 Recessive model[a]	0.245
NYHA ≥2 vs *IL18* -607 rs1946518 C>A Genotypes[b]	**0.021**
NYHA ≥2 vs *IL18* -607 rs1946518 Dominant model[a]	0.760
NYHA ≥2 vs *IL18* -607 rs1946518 Heterozygous model[a]	0.104
NYHA ≥2 vs *IL18* -607 rs1946518 Recessive model[a]	**0.009**
NYHA ≥2 vs *IL18* -137 rs187238 C>G Genotypes[b]	0.358
NYHA ≥2 vs *IL18* -137 rs187238 Dominant model[a]	1.000
NYHA ≥2 vs *IL18 -137* rs187238 Heterozygous model[a]	0.546
NYHA ≥*2* vs *IL18 -137* rs187238 Recessive model[a]	0.194
NYHA ≥2 vs Age Range[b]	0.349
NYHA ≥2 vs Sex[a]	**0.019**
NYHA ≥2 vs Digestive Involvement[a]	< **0.001**

**Cardiopathy**	
Cardiopathy vs White/Non-white[a]	0.747
Cardiopathy vs Sex	0.023
Cardiopathy vs HIV[a]	**0.045**
Cardiopathy vs *IL1B* -31 rs1143627 T>C Genotypes[b]	0.532
Cardiopathy vs *IL1B* -31 rs1143627 Dominant model[a]	0.733
Cardiopathy vs *IL1B* -31 rs1143627 Heterozygous model[a]	0.317
Cardiopathy vs *IL1B* -31 rs1143627 Recessive model[a]	0.546
Cardiopathy vs *IL6* -174 rs1800795 C>G Genotypes[b]	0.503
Cardiopathy vs *IL6* -174 rs1800795 Dominant model[a]	1.000
Cardiopathy vs *IL6* -174 rs1800795 Heterozygous model[a]	0.647
Cardiopathy vs *IL6* -174 rs1800795 Recessive model[a]	0.368
Cardiopathy vs *IL17A* -152 rs2275913 G>A Genotypes[b]	0.077
Cardiopathy vs *IL17A* -152 rs2275913 Dominant model[a]	0.883
Cardiopathy vs *IL17A* -152 rs2275913 Heterozygous model[a]	0.439
Cardiopathy vs *IL17A* -152 rs2275913 Recessive model[a]	**0.039**
Cardiopathy vs *IL18* -607 rs1946518 C>A Genotypes[b]	**0.030**
Cardiopathy vs *IL18* -607 rs1946518 Dominant model[a]	0.301
Cardiopathy vs *IL18* -607 rs1946518 Heterozygous model[a]	0.392
Cardiopathy vs *IL18* -607 rs1946518 Recessive model[a]	**0.011**

Cardiopathy vs *IL18* -137 rs187238 C>G Genotypes[b]	0.167
Cardiopathy vs *IL18* -137 rs187238 Dominant model[a]	0.670
Cardiopathy vs *IL18* -137 rs187238 Heterozygous model[a]	0.561
Cardiopathy vs *IL18* -137 rs187238 Recessive model[a]	0.078
Cardiopathy vs Age Range[b]	0.439
Cardiopathy vs Digestive Involvement[a]	**0.012**
**LVEF<45%**	
LVEF<45% vs White/Non-white[a]	1.000
LVEF<45% vs Sex[a]	0.128
LVEF<45% vs HIV[a]	**<0.001**
LVEF<45% vs *IL1B* -31 rs1143627 T>C Genotypes[b]	0.093
LVEF<45% vs *IL1B* -31 rs1143627 Dominant model[a]	0.275
LVEF<45% vs *IL1B* -31 rs1143627 Heterozygous model[a]	**0.033**
LVEF<45% vs *IL1B* -31 rs1143627 Recessive model[a]	0.198
LVEF<45% vs *IL6* -174 rs1800795 C>G Genotypes[b]	**0.034**
LVEF<45% vs *IL6* -174 rs1800795 Dominant model[a]	0.268
LVEF<45% vs *IL6* -174 rs1800795 Heterozygous model[a]	1.000
LVEF<45% vs *IL6* -174 rs1800795 Recessive model[a]	**0.028**
LVEF<45% vs *IL17A* -152 rs2275913 G>A Genotypes[b]	0.136
LVEF<45% vs *IL17A* -152 rs2275913 Dominant model[a]	0.635
LVEF<45% vs *IL17A* -152 rs2275913 Heterozygous model[a]	0.191
LVEF<45% vs *IL17A* -152 rs2275913 Recessive model[a]	0.157
LVEF<45% vs *IL18* -607 rs1946518 C>A Genotypes[b]	**0.012**
LVEF<45% vs *IL18* -607 rs1946518 Dominant model[a]	0.154
LVEF<45% vs *IL18* -607 rs1946518 Heterozygous model[a]	0.447
LVEF<45% vs *IL18* -607 rs1946518 Recessive model[a]	**0.003**
LVEF<45% vs *IL18* -137 rs187238 C>G Genotypes[b]	0.127
LVEF<45% vs *IL18* -137 rs187238 Dominant model[a]	0.446
LVEF<45% vs *IL18* -137 rs187238 Heterozygous model[a]	0.876
LVEF<45% vs *IL18* -137 rs187238 Recessive model[a]	0.052
LVEF<45% vs Age Range[b]	0.451
LVEF<45% vs Digestive Involvement[a]	**<0.001**
**Parasitemia**	
Parasitemia vs Caucasian/Non- Caucasian[a]	0.873
Parasitemia vs Cardiopathy[a]	0.885
Parasitemia vs NYHA ≥2 [a]	0.180
Parasitemia vs HIV[a]	**0.008**
Parasitemia vs *IL1B* -31 rs1143627 T>C Genotypes[b]	0.429
Parasitemia vs *IL1B* -31 rs1143627 Dominant model[a]	0.737
Parasitemia vs *IL1B* -31 rs1143627 Heterozygous model[a]	0.255
Parasitemia vs *IL1B* -31 rs1143627 Recessive model[a]	0.366
Parasitemia vs *IL6* -174 rs1800795 C>G Genotypes[b]	**0.027**
Parasitemia vs *IL6* -174 rs1800795 Dominant model[a]	0.139
Parasitemia vs *IL6* -174 rs1800795 Heterozygous model[a]	**0.023**
Parasitemia vs *IL6* -174 rs1800795 Recessive model[a]	0.145
Parasitemia vs *IL17A* -152 rs2275913 G>A Genotypes[b]	0.887
Parasitemia vs *IL17A* -152 rs2275913 Dominant model[a]	1.000
Parasitemia vs *IL17A* -152 rs2275913 Heterozygous model[a]	0.878
Parasitemia vs *IL17A* -152 rs2275913 Recessive model[a]	0.777
Parasitemia vs *IL18* -607 rs1946518 C>A Genotypes[b]	0.504
Parasitemia vs *IL18* -607 rs1946518 Dominant model[a]	0.556

Parasitemia vs *IL18* -607 rs1946518 Heterozygous model[a]	0.258
Parasitemia vs *IL18* -607 rs1946518 Recessive model[a]	0.594
Parasitemia vs *IL18* -137 rs187238 C>G Genotypes[b]	0.313
Parasitemia vs *IL18* -137 rs187238 Dominant model[a]	0.888
Parasitemia vs *IL18* -137 rs187238 Heterozygous model[a]	0.558
Parasitemia vs *IL18* -137 rs187238 Recessive model[a]	0.213
Parasitemia vs LVEF<45%[a]	**0.031**
Parasitemia vs Age Range[b]	0.326
Parasitemia vs Digestive Involvement[a]	0.438

LVEF: left ventricle ejection fraction, NIHA: New York Heart Association score.
Test: [a]Fisher exact test, [b]Pearson chi square test. P values ≤ 0.05 in bold.
