# Supplementary material for: A Specific IL6 Polymorphic Genotype Modulates the Risk of Trypanosoma cruzi Parasitemia While IL18, IL17A, and IL1B Variant Profiles and HIV Infection Protect Against Cardiomyopathy in Chagas Disease
Source: Front Immunol. 2020 Oct 22;11:521409. doi: 10.3389/fimmu.2020.521409 (PMC7642879; doi:10.3389/fimmu.2020.521409)
Supplement: Supplementary file 4 [file Table_4.pdf]

**Supplemmentary Table 4.** Skin color distribution and comparisons in Chagas cardiopathy, NYHA, LVEF and parasitemia

| | Non-white n (%) | white n (%) | P |
|---|---|---|---|
| Chagas cardiopathy: no | 23 (42.6) | 60 (39.5) | 0.747 |
| Chagas cardiopathy: yes | 31 (57.4) | 92 (60.5) | |
| NYHA <2 /No CA | 29 (59.2) | 83 (58.5) | 1.000 |
| NYHA≥2 | 20 (40.8) | 59 (41.5) | |
| Missing | 5 | 10 | |
| LVEF≥45% | 30 (62.5) | 85 (62.5) | 1.000 |
| LVEF<45% | 18 (37.5) | 51 (37.5) | |
| Missing | 6 | 16 | |
| Parasitemia: no | 29 (53.7) | 83 (56.1) | 0.873 |
| Parasitemia: yes | 25 (46.3) | 65 (43.9) | |
| Missing | 0 | 4 | |

LVEF: left ventricular ejection fraction. NYHA: New York Heart Association score. No CA – without cardiopathy. Comparisons were performed by Fisher exact test. Percentual distributions considered only valid cases. When missing values were not shown those values are zero.
