# Supplementary material for: A Specific IL6 Polymorphic Genotype Modulates the Risk of Trypanosoma cruzi Parasitemia While IL18, IL17A, and IL1B Variant Profiles and HIV Infection Protect Against Cardiomyopathy in Chagas Disease
Source: Front Immunol. 2020 Oct 22;11:521409. doi: 10.3389/fimmu.2020.521409 (PMC7642879; doi:10.3389/fimmu.2020.521409)
Supplement: Supplementary file 5 [file Table_5.pdf]

Supplemmentary Table 5. Haplotype analyses for *IL18* rs1946518 and rs187238

Chagas Cardiopathy

Haplotype	Total haplotype frequency	With Chagas Cardiopathy		No Chagas Cardiopathy		With Chagas Cardiopathy haplotype frequency	No Chagas Cardiopathy haplotype frequency	P
		Haplotype count	Other haplotypes	Haplotype count	Other haplotypes			
CC	0.568	151.3	94.7	82.7	83.3	0.615	0.498	**0.0186**
AG	0.257	58.3	187.7	47.7	118.3	0.237	0.287	0.2534
AC	0.158	32.7	213.3	32.3	133.7	0.133	0.195	0.0911
CG	0.017	3.7	242.3	3.3	162.7	0.015	0.020	0.6977

NYHA

Haplotype	Total haplotype frequency	NYHA≥2		No CA or NYHA<2		NYHA≥2 haplotype frequency	No CA or NYHA<2 frequency	P
		Haplotype count	Other haplotypes	Haplotype count	Other haplotypes			
CC	0.568	98.5	59.5	117.6	106.4	0.623	0.525	0.0554
AG	0.257	37.5	120.5	59.6	164.4	0.237	0.266	0.5286
AC	0.158	19.5	138.5	42.4	181.6	0.123	0.189	0.0843
CG	0.017	2.5	155.5	4.4	219.6	0.016	0.020	0.7701

LVEF

Haplotype	Total haplotype frequency	LVEF<45%		LVEF≥45%		LVEF>45% haplotype frequency	LVEF≥45% haplotype frequency	P
		Haplotype count	Other haplotypes	Haplotype count	Other haplotypes			
CC	0.568	89.6	48.4	119.5	110.5	0.649	0.519	**0.0149**
AG	0.257	27.6	110.4	65.5	164.5	0.200	0.285	0.0707
AC	0.158	17.4	120.6	41.5	188.5	0.126	0.181	0.1672
CG	0.017	3.4	134.6	3.5	226.5	0.025	0.015	0.529

Parasitemia

Haplotype	Total haplotype frequency	Parasitemia: positive		Parasitemia: negative		Parasitemia: positive haplotype frequency	Parasitemia: negative haplotype frequency	P
		Haplotype count	Other haplotypes	Haplotype count	Other haplotypes			
CC	0.568	104.5	75.5	124.5	99.5	0.581	0.556	0.6124
AG	0.257	51.5	128.5	52.5	171.5	0.286	0.234	0.2338
AC	0.158	22.5	157.5	41.5	182.5	0.125	0.185	0.097
CG	0.017	1.5	178.5	5.5	218.5	0.008	0.025	0.2031

LVEF: left ventricle ejection fraction, NYHA: New York heart association score. No CA – without cardiopathy. All haplotype counts and frequencies were estimated using Haploview, based on the genotype distribution observed in the analyzed population. P values ≤ 0.05 in bold.
